# Causal Relationship between Gut Microbiota and Endometrial Cancer: A Two-Sample Mendelian Randomization Study

**DOI:** 10.7150/ijms.112922

**Published:** 2025-06-23

**Authors:** Xinrui Yao, Sitong Dong, Xiao Li, Ouxuan Liu, Yuexin Hu, Yuxuan Wang, Xiangcheng Fan, Bei Lin

**Affiliations:** 1 Department of Obstetrics and Gynecology, Shengjing Hospital of China Medical University, No. 36, Sanhao Street, Heping District, Shenyang, 110004, China.; 2 Key Laboratory of Maternal-Fetal Medicine of Liaoning Province, Key Laboratory of Obstetrics and Gynecology of Higher Education of Liaoning Province, Shenyang, China.; 3 Center of Reproductive Medicine, Shengjing Hospital of China Medical University, No. 39 Huaxiang Road, Shenyang 110004, China.; 4 Shenyang Reproductive Health Clinical Medicine Research Center, Shenyang 110004, China.

**Keywords:** Mendelian randomization, gut microbiota, endometrial cancer, causality, genetics

## Abstract

Several relevant reports have shown that changes in the composition of the gut microbiota are related to the pathogenesis of endometrial cancer (EC). However, the causal effect of the gut microbiota on EC remains unknown. A two-sample Mendelian randomization (MR) study was used to assess the causal effects of the gut microbiota on EC, EC with endometrioid histologies and EC with non-endometrioid histologies. The genetic statistics of the gut microbiota, including 18,340 participants, were acquired from the MiBioGen database. The summary statistics of EC, EC with endometrioid histologies and EC with non-endometrioid histologies were obtained from the publicly available Genome-wide Association Study (GWAS) database. Suitable single nucleotide polymorphisms (SNPs) were selected as instrumental variables (IVs) (*P* < 5×10^-8^, r^2^ < 0.001). The causal effects were evaluated via the MR-Egger regression method, the inverse-variance weighted (IVW) method, the weighted median test, the weighted mode test, and the simple mode test. The IVW analysis suggested that *Ruminococcusgnavusgroup* (OR=0.82, 95%CI=0.78-0.85, *P*=1.29×10^-17^), *Euryarchaeota* (OR=0.90, 95%CI=0.87-0.94, *P*=3.78×10^-6^), and *CandidatusSoleaferrea* (OR=0.92, 95%CI=0.87-0.98, *P*=0.01) had protective effects on EC and its subtypes. *Gammaproteobacteria* (OR=1.29, 95%CI=1.19-1.39, *P*=2.32×10^-10^) served as a risk factor for EC, and *Intestinimonas* (OR=1.33, 95%CI=1.10-1.62, *P*=3.68×10^-3^) had detrimental effects on EC with non-endometrioid histologies. The causal relationship between the gut microbiota and EC was explored through two-sample MR analysis, which is helpful for further understanding the gut microbiota-mediated development mechanism underlying EC.

## Introduction

Endometrial cancer (EC) is one of the most common gynecological cancers worldwide, and is an epithelial malignancy that occurs in the endometrium. Recently, the incidence of EC has been increasing globally 1. The most common symptom of EC is postmenopausal bleeding. Although the treatment of EC is still challenging, understanding various pathogenic state drivers of this disease and genetic diversity has led to the development of different treatment approaches to improve the precision of this complex malignancy 2. Histologically, two ECs with morphological and molecular differences and therapeutic implications have been identified. The occurrence of type I EC is directly related to the continuous stimulation of estrogen without progesterone antagonism. In the absence of progesterone antagonism, the endometrium is in a state of hyperplasia for a long period of time and further develops into EC. Type II EC shows non-endometrioid differentiation and follows an estrogen-free pathway, and the mechanism is not fully understood 3. The main risk factors for EC are obesity, diabetes mellitus, hypertension, and reproductive endocrine disorders, such as polycystic ovarian syndrome (PCOS) 2. The prevention of risk factors can reduce the occurrence and development of EC to a certain extent.

The gut microbiota is a complex microbial population in the human gastrointestinal tract with important effects on homeostasis and disease in the host. The gut microbiota plays a crucial role in protecting against pathogens and maintaining immune and metabolic homeostasis. An increasing number of studies suggest that the gut microbiota is not only a central regulator of various inflammatory and proliferative diseases but also essential for physiological gastrointestinal function. Changes in the gut bacterial composition are implicated in the pathogenesis of many inflammatory diseases and infections 4. Current evidence suggests that the composition of the gut microbiota is significantly different between EC patients and controls. Compared with those in the normal group, the abundances of *Bacteroidota* and *Verrucomicrobiota* increased in the EC group, whereas that of *Proteobacteria* decreased in the EC group 5. The gut microbiota affects the metabolism of estrogen by secreting beta-glucuronidase (GUS), an enzyme that deconjugates estrogen to estrogen binding receptors, which indirectly influences the occurrence and development of EC 6. However, most previous studies on the gut microbiota and cancer are case control studies, whose results are difficult to determine. Furthermore, the relationships between the gut microbiota and disease are susceptible to the environment, lifestyle, age, dietary patterns and other confounding factors, which cannot be avoided in observational studies. The causal inference of the gut microbiota and EC is limited by these conditions.

Mendelian randomization (MR) is a new epidemiological method based on whole-genome sequencing data, and single nucleotide polymorphisms (SNPs) were chosen as instrumental variables (IVs) to reveal causality 7. Compared with observational studies such as cohort studies, MR is less affected by reverse causality and confounding factors, thus effectively reducing bias 8. MR requires the satisfaction of three important hypotheses: (1) In the relevance hypothesis, IVs should be closely related to exposure factors. (2) In the independence hypothesis, the IV should be independent of any confounders and should be independent of whether these confounding factors can be observed. (3) Exclusivity hypothesis, IVs mediate outcomes only via exposure factors, not other pathways. At present, MR analysis is used for the causal exploration of gut microbiota and a variety of diseases, such as autoimmune diseases 9, heart failure 10, and primary liver cancer 11. However, there are no published MR studies of the gut microbiota and EC.

This study conducted a two-sample MR analysis between the gut microbiota and EC, aiming to explore this association, to provide new theoretical support for further understanding the occurrence and development of EC.

## Materials and Methods

### Study design

**Figure 1:**
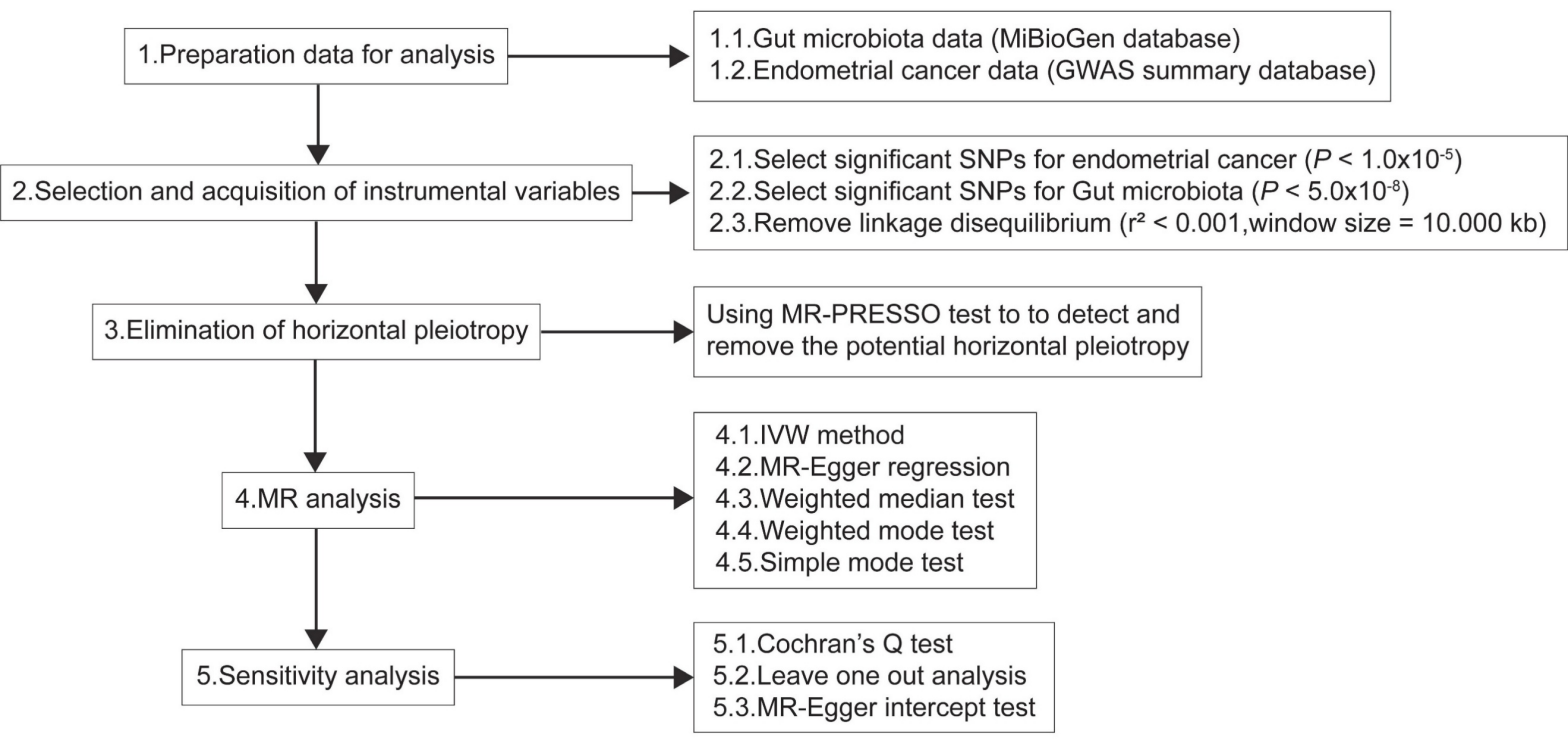
Diagrammatic description of MR analysis. IVW, inverse-variance weighted; PRESSO, Pleiotropy RESidual Sum and Outlier; MR, Mendelian randomization; SNP, single nucleotide polymorphism.

The causal relationship between the gut microbiota and EC was evaluated on the basis of statistics from two MR datasets. **Figure 1** shows the flowchart of the study design.

### Data source

The Genome-wide Association Study (GWAS) statistics of EC (12,906 cases and 108,979 controls, 9,470,555 SNPs), EC with endometrioid histologies (8,758 cases and 46,126 controls, 9,464,330 SNPs) and EC with non-endometrioid histologies (1,230 cases and 35,447 controls, 8,974,630 SNPs) were downloaded as outcomes from the Open GWAS database. According to pathology reports, the histological subtype of EC was confirmed 12, 13. The genetic statistics of the gut microbiota were obtained from the MiBioGen database, a large-scale multi-ethnic GWAS for 16S rRNA gene sequencing data collected from 24 cohorts including approximately 18,340 individuals 14. A total of 211 taxa (9 phyla, 16 classes, 20 orders, 35 families and 131 genera) identified via microbiota quantitative trait locus (mbQTL) mapping analysis were included. After excluding 12 unknown genera, 119 taxa were included for analysis.

### Acquisition of instrumental variables (IVs)

According to the GWAS data and IVs screened out from the previous step, the outcome-related IVs were removed at the locus-wide significant threshold (*P* < 1.0×10^-5^). The IVs (SNPs) that were sensibly associated with exposure factors were identified via the extract instrument function of the R package TwoSampleMR (version 0.5.6) 15 By setting *P* < 5×10^-8^, IVs with linkage disequilibrium (LD) were removed via clump = TRUE. On the basis of European ancestry reference data from the 1000 Genomes Project, the SNPs with LD (r^2^ < 0.001 and clumping window size = 10,000 kb) were excluded. Potential horizontal pleiotropy was detected using the MR pleiotropy residual sum and outlier (MR-PRESSO) test, and the effect of pleiotropy was eliminated by removing outliers 16. Eventually, the effect sizes and alleles of the SNPs on the exposure and outcome data were harmonized, and the incompatible alleles were excluded.

### Statistical analysis

In this study, the “TwoSampleMR” R package 15 was used for two-sample MR analysis between exposure and outcome, and five common MR methods were used for features that contained more than one IV: simple mode test, weighted mode test, weighted median test, MR-Egger regression, and inverse-variance weighted (IVW) method. The IVW test is the main method for studying the causal relationship between the gut microbiota and EC. The IVW method was first proposed by Burgess *et al.* 17 for MR studies with multiple IVs. This method was used to calculate the Wald ratio for each IV and to combine the outcome with the use of IVW analysis. Each IV was weighted according to the inverse of the effect variance. Thus, large studies with smaller standard errors can gain more weight than small studies with larger standard errors. This weight choice can minimize imprecision in the pooled effect estimates. The slope of the IVW method can be interpreted as the causal effect of exposure on the outcome. The fixed or multiplicative random effects model can be used to estimate the variance of the effect. Bowden *et al.* 18 proposed the MR-Egger method , which calculates the Wald ratio for each IV and combines the outcomes via Egger regression. Egger regression can be used to test for pleiotropy bias, and its slope coefficient reflects the size of the causal effect. It can provide a valid test for the null hypothesis of causality and unbalanced directional pleiotropy. The weighted median test calculates the Wald ratio for each instrument, and the median can be selected as the causal estimate variable. This method does not rely on Instrument Strength Independent of Direct Effect (InSIDE) assumptions and provides reliable causal estimates despite having fewer than 50% valid SNPs 19. The calculation process of the simple model is roughly the same as that of a weighted model method, that is, the causal effect estimates of individual IVs are clustered. Then, the causal effect estimate is calculated for the largest set of IVs 20, but the weighted model method assigns the weight to each IV. To ensure the accuracy of the MR results, sensitivity analysis was performed. The presence of heterogeneity was tested via Cochran's Q test, and there was no significant heterogeneity at a *P* value greater than 0.05. Whether the pooled estimation was biased by single SNPs was appraised via the leave-one-out analysis. This method calculates the overall effect of the remaining IVs by gradually eliminating each IV and observing whether the outcome changes after eliminating each IV. If the outcome changed significantly after excluding a variable, it indicated that the variable might be an invalid SNP or have a special effect on the outcome. Horizontal pleiotropy was detected via the MR-Egger intercept test. Under the InSIDE hypothesis, the intercept of the line fitted by the MR-Egger regression intercept reflects horizontal pleiotropy, and there is horizontal pleiotropy of MR if the intercept is not equal to zero.

## Results

### Two-sample MR analysis

After a series of quality selective steps, 270 SNPs related to six microbiota features for EC, including genus *CandidatusSoleaferrea*, genus *Ruminococcusgnavusgroup*, phylum *Euryarchaeota*, class. *Gammaproteobacteria*, genus *Eubacteriumeligensgroup* and genus *Intestinimonas* were identified via at least one MR method. The screening results of specific SNPs are shown in **Supplementary Tables 1-3**.

**Figure 2:**
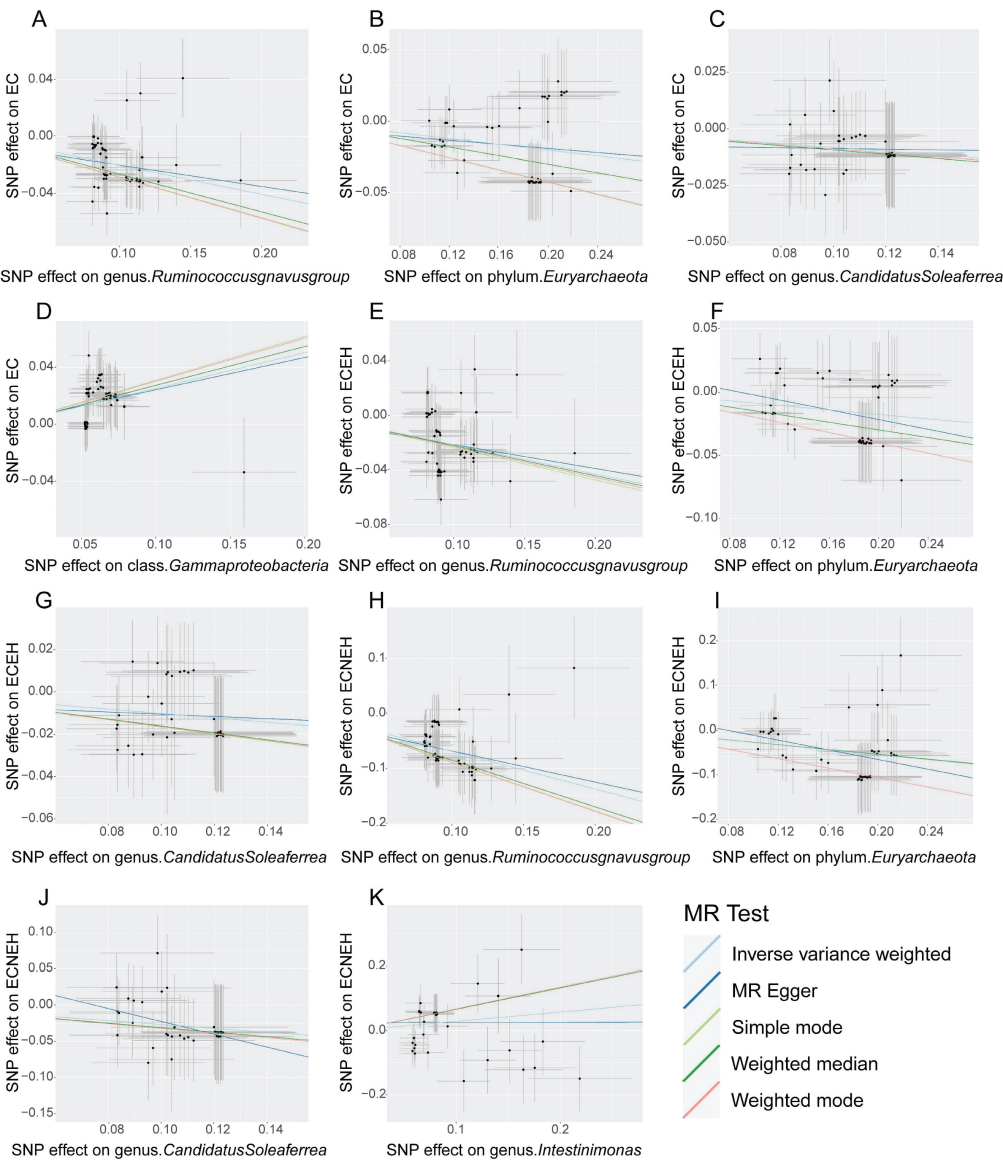
The causality of gut microbiota and EC. (**A**) The causal effect of *Ruminococcusgnavusgroup* on EC. (**B**) The causal effect of *Euryarchaeota* on EC. (**C**) The causal effect of *CandidatusSoleaferrea* on EC. (**D**) The causal effect of *Gammaproteobacteria* on EC. (**E**) The causal effect of *Ruminococcusgnavusgroup* on ECEH. (**F**) The causal effect of *Euryarchaeota* on ECEH. (**G**) The causal effect of *CandidatusSoleaferrea* on ECEH. (**H**) The causal effect of *Ruminococcusgnavusgroup* on ECNEH. (**I**) The causal effect of *Euryarchaeota* on ECNEH. (**J**) The causal effect of* CandidatusSoleaferrea* on ECNEH. (**K**) The causal effect of *Intestinimonas* on ECNEH. EC, endometrial cancer; ECEH, endometrial cancer with endometrioid histologies; ECNEH, endometrial cancer with non-endometrioid histologies.

On the basis of the IVW results (**Table 1**), *Ruminococcusgnavusgroup* (OR=0.82, 95%CI=0.78-0.85, *P*=1.29×10^-17^), *Euryarchaeota* (OR=0.90, 95%CI=0.87-0.94, *P*=3.78×10^-6^), *CandidatusSoleaferrea* (OR=0.92, 95%CI=0.87-0.98, *P*=0.01) and *Gammaproteobacteria* (OR=1.29, 95%CI=1.19-1.39, *P*=2.32×10^-10^) had significant causal relationships with EC, while *Eubacteriumeligensgroup* and *Intestinimonas* had no causal effect with EC. These findings suggested that *Ruminococcusgnavusgroup*, *Euryarchaeota* and *CandidatusSoleaferrea* were related to the decreased risk of EC, and *Gammaproteobacteria* was related to the increased risk of EC. In the secondary analysis, *Ruminococcusgnavusgroup*, *Euryarchaeota* and *CandidatusSoleaferrea* had preventive effects in EC with endometrioid histologies and EC with non-endometrioid histologies, while *Intestinimonas* (OR=1.33, 95%CI=1.10-1.62, *P*=3.68×10^-3^) had a positive effect on the risk of EC with non-endometrioid histologies. As shown in **Supplementary Figures 1-3**, the forest plot showed that the IVW results for *Ruminococcusgnavusgroup*, *Euryarchaeota*, *CandidatusSoleaferrea* were less than 0, indicating that these three microorganisms were preventive factors for EC, EC with endometrioid histologies and EC with non-endometrioid histologies. *Gammaproteobacteria* was a risk factor for EC and *Intestinimonas* was positively related to the risk of EC with non-endometrioid histologies. Moreover, four other methods were also used to test the MR, and the results were consistent with the results estimated by the IVW model in terms of magnitude and direction (**Table 2**). **Figure 2** presented a scatter plot of the causal relationship between the gut microbiota and EC, the results of which further confirmed that all methods have a stable direction without the occurrence of outliers.

### Heterogeneity and sensitivity analysis

**Figure 3:**
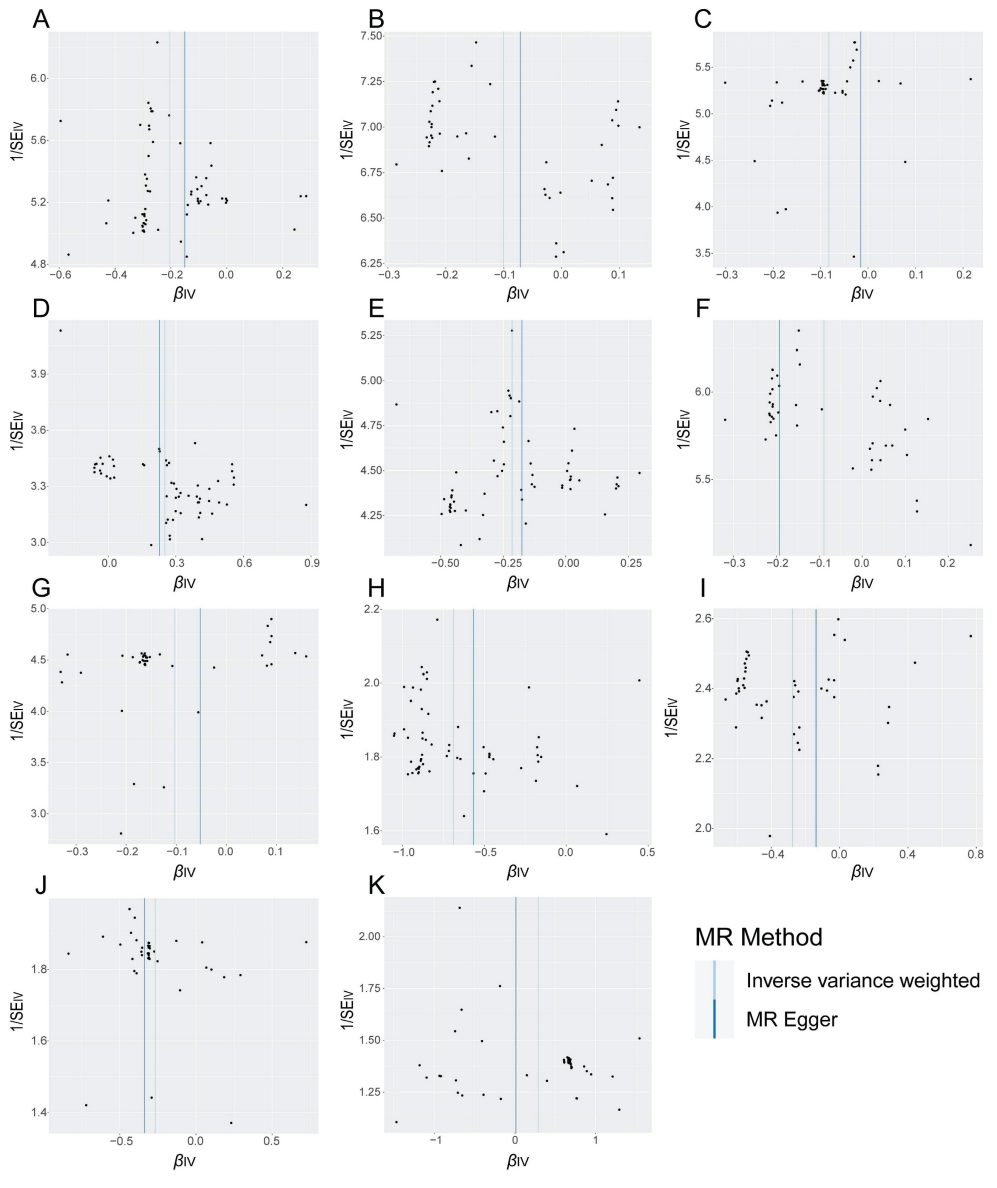
The judgment of randomness of Mendel's second Law. (**A**) Random judgment of MR analysis for SNPs of *Ruminococcusgnavusgroup* and EC. (**B**) Random judgment of MR analysis for SNPs of *Euryarchaeota* and EC. (**C**) Random judgment of MR analysis for SNPs of *CandidatusSoleaferrea* and EC. (**D**) Random judgment of MR analysis for SNPs of *Gammaproteobacteria* and EC. (**E**) Random judgment of MR analysis for SNPs of *Ruminococcusgnavusgroup* and ECEH. (**F**) Random judgment of MR analysis for SNPs of *Euryarchaeota* and ECEH. (**G**) Random judgment of MR analysis for SNPs of *CandidatusSoleaferrea* and ECEH. (**H**) Random judgment of MR analysis for SNPs of *Ruminococcusgnavusgroup* and ECNEH. (**I**) Random judgment of MR analysis for SNPs of *Euryarchaeota* and ECNEH. (**J**) Random judgment of MR analysis for SNPs of *CandidatusSoleaferrea* and ECNEH. (**K**) Random judgment of MR analysis for SNPs of *Intestinimonas* and ECNEH. MR, Mendelian randomization; SNP, single nucleotide polymorphism; EC, endometrial cancer; ECEH, endometrial cancer with endometrioid histologies; ECNEH, endometrial cancer with non-endometrioid histologies.

Funnel plot is a subjective visualization method to assess heterogeneity based on the distribution of individual SNPs, which provides the distribution and strength of each genetic variant in the association between the gut microbiota and EC. The funnel diagram (**Figure 3**) showed that the SNPs were randomly distributed on both sides of the IVW line, indicating that Mendel's second law was followed. Meanwhile, the results of the heterogeneity test showed that all *P* values were greater than 0.05 (**Table 3**), indicating that there was no significant heterogeneity in this study. The magnitude of assessed level pleiotropy was tested by MR Egger intercept analysis, and the results of the pleiotropy test are shown in **Table 4**. The MR Egger intercepts of the MR pleiotropy tests of EC and *CandidatusSoleaferrea* (*P*=0.7598), *Ruminococcusgnavusgroup* (*P*=0.7342), *Euryarchaeota* (*P* =0.7304) and *Gammaproteobacteria* (*P*=0.9204) were -0.0072, -0.0051, -0.0047 and 0.0015, respectively. In addition, there was no evidence of pleiotropy detected by the MR Egger analysis between the two subtypes of EC (endometrioid EC and non-endometrioid EC) and the gut microbiota (all *P* > 0.05). Sensitivity analyses were then performed with the use of the Leave-one-out method. Single SNP was eliminated one by one, and the remaining SNPS were re-analyzed by IVW method. Leave-one-out sensitivity tests demonstrated that the overall results were consistent and there were no SNPs with extremely high sensitivity. Oversensitivity to individual SNP loci according to the of MR results was not exhibited (**Supplementary Figures 4-6**). In summary, the MR results were reliable.

## Discussion

Although traditional observational studies are trying to investigate the role of the gut microbiota in diseases, the defects of the method itself are that it is easily affected by many confounding factors. Therefore, the direct and exact causal relationships between the gut microbiota and diseases cannot be elucidated. On the basis of the GWAS data, a two-sample MR analysis was conducted to obtain instrumental variable information on exposure and outcome, and its sensitivity was tested, with the goal of identifying evidence of the causal relationship between gut microbiota and EC. Since IVs are not affected by the reverse causality of traditional epidemiological studies and confounding factors, reliable evidence can be provided to support the correlation between gut microbiota and EC. These results indicated that *Gammaproteobacteria* were associated with the risk of EC. Among the histological subtypes of EC (EC with endometrioid histologies and EC with non-endometrioid histologies), the direction of risk estimation for *Gammaproteobacteria* was consistent with that for EC (although the results of IVW were not significant). *Euryarchaeota*, *CandidatusSoleaferrea* and *Ruminococcusgnavusgroup* may reduce the risk of EC.

Approximately 10^^14^ microorganisms colonize the human intestinal tract; these microorganisms are diverse and have important functions such as promoting food digestion and absorption, maintaining the stability of the internal environment, protecting the intestinal mucosal barrier, and regulating metabolism and immunity. Disorders of the gut microbiota can cause pathological changes in the body, leading to the occurrence of chronic diseases such as obesity, diabetes, metabolic diseases and cardiovascular diseases. Moreover, the gut microbiota plays an irreplaceable role in the occurrence and development of tumors. Type I EC, representing 80-85% of the total incidence of EC, is mainly associated with monoestrogenic increase without progestogen opposition. Estrogen has a long-term effect on the endometrium, which can increase the proliferation, migration and invasion of endometrial cells, and then induce endometrial atypical hyperplasia and deterioration. Estrogen metabolism mainly occurs in the liver, where it is glucuronidated and sulfonated to form inactive glucuronide-estrogen conjugates, which are excreted into the intestine through bile and bound to GUS produced by intestinal bacteria. Estrogen exposure is caused by reabsorption into the circulation in the form of active free estrogen through the intestinal mucosa. It has been found that *Bacteroides*, *Bifidobacterium*, *Collinella*, *Aliella*, *Edwardiella*, *Faecalibacterium*, *Lactobacillus* and *Rosberia* can encode GUS and increase estrogen in the body 21. Moreover, the gut microbiota is highly correlated with EC risk factors such as obesity, diabetes, and PCOS. Obesity further changes the abundance of intestinal microorganisms and affects the synthesis of estrogen. An increase of estrogen promotes the inflammatory response in the body. The increased inflammatory factors stimulate the synthesis of estrogen in the body by participating in the synthesis of aromatase and 17β-hydroxysteroid dehydrogenase, forming an interactive multi-pathway stimulation loop and increasing the risk of EC 3.

Yue *et al.* 22 demonstrated that the abundance of *Gammaproteobacteria* in EC patients was greater than that in the control group through 16S rRNA high-throughput gene sequencing. As an important group of *Proteobacteria*, *Gammaproteobacteria* is parasitic in the mucosa adjacent to the intestinal lumen and remains at a low level in the healthy intestine. The increase of its abundance is often associated with intestinal flora disorder, intestinal dysfunction and inflammation 23. The intestinal barrier is disrupted by disorders of the gut microbiota and intestinal inflammation. Pathogenic factors in the intestinal lumen can enter the blood circulation via the damaged intestinal barrier reach distant organs, and thereby induce EC 24-27. Lipopolysaccharide (LPS) and trimethylamine N-oxide (TMAO) are the main functional factors of *Gammaproteobacteria*. In addition to being a major component of the cell wall of Gram-negative bacteria, LPS is also a pathogen-associated molecular pattern (PAMP) and inflammatory inducer. Several studies have demonstrated that LPS can promote tumor progression via inflammatory and metabolic pathways. For instance, Jiang *et al.* 28 revealed that LPS affect cervical cancer cell proliferation and glucose metabolism by modulating the FRA1/MDM2/p53 pathway. In breast cancer, LPS has been shown to induce inflammation through the prostaglandin E2 (PGE2)-EP2 signaling pathway, thereby facilitating pulmonary metastasis 29. TMAO, a gut microbiota-derived metabolite associated with increased cardiovascular risk, has also garnered attention for its potential role in tumorigenesis. Zhou *et al.* 30 demonstrated through *in vivo* and *in vitro* experiments that TMAO promotes hepatocellular carcinoma cell proliferation, migration, and epithelial-mesenchymal transition (EMT) by activating the MAPK pathway. Yang *et al.* 31 found that TMAO stimulates colorectal cancer cell proliferation and elevates vascular endothelial growth factor A (VEGFA) levels, further driving cancer progression. These findings suggest that LPS and TMAO may similarly influence EC progression through analogous mechanisms, though further experimental validation is required. Additionally, some studies have found that compared with normal control populations, EC-related metabolic diseases, including PCOS and type 2 diabetes mellitus (T2DM) patients, have intestinal barrier dysfunction and the content of *Gammaproteobacteria* in feces was significantly increased 32, 33, and hence it can be speculated that *Gammaproteobacteria* indirectly cause EC by promoting EC high-risk factors through its functional factors. Some studies have also shown that the peripheral LPS and TMAO levels are much higher in PCOS and T2DM patients than in control individuals, which further supports our speculation 34-37.

This study revealed that *CandidatusSoleaferrea* exerted preventive effects on EC the occurrence and development and that the reason may be related to glucagon-like peptide 2 (GLP-2). According to Cai *et al.* 38, the abundance of *CandidatusSoleaferrea* had a positive correlation with the level of GLP-2, a trophic hormone secreted by intestinal endocrine L cells in response to stimulation with short-chain fatty acids (SCFAs), a type of metabolite of the gut microbiota, that is involved in maintaining the morphology and function of the intestinal epithelium and improving the intestinal mucosal barrier. It can enhance the immune defense function, reduce the amount of LPS released from the intestinal barrier into the circulation, and exert the anti-tumor effect 39. In addition, some studies have found that GLP-2 regulates hepatic glucose metabolism in mice through the activation of the GLP-2R-PI3K-Akt-FoxO1 signaling pathway, and mice that lack GLP-2 receptors show glucose intolerance and hepatic insulin resistance (IR), which means that GLP-2 contributes greatly to the control of glucose homeostasis and insulin sensitivity 40. Therefore, it is speculated *CandidatusSoleaferrea* may exert a protection impact within EC by inhibiting inflammation and IR through GLP-2. By analyzing the symbiotic flora in the human normal colon, adjacent colorectal cancer and colon tumor tissues, Zhang *et al.* 41 found that normal colon tissues were rich in *Ruminococcus gnavus*. This inhibited the growth of colon tumors and promoted the immune surveillance function of CD8^+^T cells by degrading lysophospholipid in mice with normal immune function. Moreover, *Ruminococcus gnavus* can increase the number of regulatory T cells in mesenteric lymph nodes and the concentration of butyric acid in the cecum of mice 42. As a beneficial metabolite produced by the gut microbiota, butyrate has been shown to inhibit tumor proliferation and promote the anticancer efficacy of chemotherapy. For example, *in vitro* experiments demonstrated that butyrate can strongly inhibit the proliferation of pancreatic cancer cells, enhance the sensitivity of pancreatic cancer cells to chemotherapeutic drugs, and promote gemcitabine-mediated tumor growth inhibition mainly by inducing apoptosis. An animal experiment revealed that butyrate can regulate the tumor microenvironment, reduce the levels of tumor extracellular matrix and macrophage markers in a mouse model of pancreatic ductal adenocarcinoma, and improve serum lipid metabolism, thus playing a tumor suppressive role 43. However, studies have shown that the abundance of *Ruminococcus gnavus* is increased in the feces of patients with Crohn's disease, inflammatory bowel disease and metabolic diseases, including obesity, T2DM and gestational diabetes mellitus, which suggested that *Ruminococcus gnavus* is pathogenic 44. Although functional metabolites of *Ruminococcus gnavus* have been identified, such as SCFAs, anti-inflammatory capsular polysaccharides and secondary bile acids, the contradictory reasons for the pathogenic or beneficial effects of *Ruminococcus gnavus* have not been elucidated. Additionally, the mechanism of the interaction between *Ruminococcus gnavus* and EC needs to be further explored. Contrary to the effects of *Gammaproteobacteria* mentioned above, *Euryarchaeota* in the human gut have the genetic potential to take hydrogen to reduce trimethylamine (TMA) and TMAO, and were found to be associated with lower fecal TMA concentrations 45. Therefore, it is hypothesized that *Euryarchaeota* could prevent metabolic disorders and malignant tumors by eliminating TMA and TMAO before it enters the circulation.

The research is among the first to explore the causal relationships between the gut microbiota and EC and its subtypes, which has the following important implications. First, SNPs obtained following the large-scale GWAS data are closely related to exposure factors, but not to outcomes, and therefore the impact of exposure factors on outcomes can be represented by the association effect between SNPs and outcomes. Due to the fact that alleles abide by the random assignment principle, this effect is not influenced by the confounding factors within the traditional observational research and can provide robust causal evidence. Second, given the paucity of data on the connection between the gut microbiota and EC, the research expands the current evidence on the causal relationship between the gut microbiota and EC. Third, the research assessed MR through multiple complementary sensitivity analyses to ensure the rigor of the study.

However, it is necessary to consider several limitations of this research. First of all, the GWAS database here was dominated by participants of European ancestry, and it remains uncertain whether the research results can be generalized to individuals of non-European ancestry because genetic differences exist between ethnic groups. In the future, we plan to actively collect multi-center, multi-regional clinical samples and data, incorporating diverse ethnic populations to reduce data heterogeneity and enhance the generalizability of findings. Second, since data on exposure factors were available only at the genus level, the connection between the gut microbiota and EC at the species level could not be analyzed. Metagenomic sequencing technologies will be employed to obtain species-level gut microbial profiles, enabling replication of MR analyses at the microbial species level. Third, although MR can reduce the influence of confounding factors, it cannot entirely eliminate the possibility that alterations in gut microbiota are a consequence rather than a cause of EC. Current basic research methodologies and techniques for the role of gut microbiota in disease pathogenesis are becoming increasingly improving. For instance, studies utilizing animal and cellular experiments have demonstrated the influence of specific gut bacterial taxa on tumorigenic behaviors 46, 47. Furthermore, longitudinal cohort studies enable the tracking of temporal dynamics, thereby helping to determine whether the gut microbiota acts as a causal driver in the pathogenesis and progression of diseases rather than a consequence 48, 49. To further substantiate the causal directionality of the associations discussed in this research, we will conduct foundational experiments (e.g., microbiota colonization experiments) and longitudinal cohort studies (e.g., monitoring microbial dynamics prior to EC onset) in future work, thereby providing more robust empirical evidence for our findings and enhancing the validity of this research.

## Conclusions

In conclusion, this study demonstrates a potential causal association between the gut microbiota and EC. Future research will investigate the specific mechanism through which the gut microbiota (*Gammaproteobacteria*, *CandidatusSoleaferrea*, *Ruminococcusgnavusgroup* and *Euryarchaeota*) affects the occurrence and development of EC.

## Supplementary Material

Supplementary figures.

Supplementary table 1.

Supplementary table 2.

Supplementary table 3.

## Figures and Tables

**Table 1:** Results of MR analysis using IVW model.

Bacterial taxa (exposure)	No. SNP	EC		ECEH		ECNEH	
		OR (95% CI)	*P* value	OR (95% CI)	*P* value	OR (95% CI)	*P* value
*CandidatusSoleaferrea*	41	0.92 (0.87-0.98)	**0.01**	0.90 (0.84-0.97)	**3.63E-03**	0.77 (0.65-0.90)	**1.77E-03**
*Ruminococcusgnavusgroup*	63	0.82 (0.78-0.85)	**1.29E-17**	0.81 (0.76-0.85)	**1.93E-14**	0.50 (0.44-0.57)	**7.46E-24**
*Euryarchaeota*	44	0.90 (0.87-0.94)	**3.78E-06**	0.91 (0.87-0.96)	**5.57E-04**	0.76 (0.67-0.86)	**1.30E-05**
*Gammaproteobacteria*	58	1.29 (1.19-1.39)	**2.32E-10**	1.07 (0.97-1.17)	0.16	1.93 (1.54-2.41)	0.76
*Eubacteriumeligensgroup*	12	1.10 (0.93-1.29)	0.26	1.22 (1.00-1.48)	0.05	1.17 (0.62-2.19)	0.63
*Intestinimonas*	52	1.05 (0.98-1.12)	0.20	0.99 (0.91-1.07)	0.76	1.33 (1.10-1.62)	**3.68E-03**

No. SNP, the number of SNPs as instrumental variables; IVW, inverse-variance weighted; MR, Mendelian randomization; EC, endometrial cancer; ECEH, endometrial cancer with endometrioid histologies; ECNEH, endometrial cancer with non-endometrioid histologies; OR, odds ratio; CI, confidence interval.

**Table 2:** Mendelian randomization analysis results using the MR-Egger regression, the weighted median test, the weighted mode test and the simple mode test.

Traits (outcome)	Bacterial taxa (exposure)	MR methods	No. SNP	OR (95% CI)	*P* value
EC	*CandidatusSoleaferrea*	MR Egger	41	0.98 (0.64-1.52)	0.94
		Weighted median		0.91 (0.84-0.98)	**0.01**
		Simple mode		0.92 (0.78-1.07)	0.29
		Weighted mode		0.92 (0.78-1.07)	0.27
	*Ruminococcusgnavusgroup*	MR Egger	63	0.86 (0.63-1.17)	0.35
		Weighted median		0.77 (0.72-0.82)	**1.06E-15**
		Simple mode		0.75 (0.65-0.86)	**1.74E-04**
		Weighted mode		0.75 (0.64-0.88)	**6.16E-04**
	*Euryarchaeota*	MR Egger	44	0.93 (0.79-1.10)	0.41
		Weighted median		0.86 (0.81-0.91)	**7.27E-07**
		Simple mode		0.81 (0.69-0.94)	**0.01**
		Weighted mode		0.81 (0.69-0.95)	**0.01**
	*Gammaproteobacteria*	MR Egger	58	1.25 (0.77-2.04)	0.36
		Weighted median		1.31 (1.18-1.46)	**6.58E-07**
		Simple mode		1.36 (1.05-1.76)	**0.02**
		Weighted mode		1.35 (1.07-1.71)	**0.02**
	*Eubacteriumeligensgroup*	MR Egger	12	1.60 (0.88-2.91)	0.15
		Weighted median		1.10 (0.89-1.37)	0.37
		Simple mode		1.28 (0.87-1.89)	0.24
		Weighted mode		1.29 (0.89-1.88)	0.21
	*Intestinimonas*	MR Egger	52	1.21 (0.95-1.54)	0.13
		Weighted median		1.07 (0.98-1.18)	0.14
		Simple mode		1.08 (0.89-1.30)	0.45
		Weighted mode		1.08 (0.90-1.29)	0.44
ECEH	*CandidatusSoleaferrea*	MR Egger	41	0.95 (0.57-1.57)	0.84
		Weighted median		0.85 (0.78-0.93)	**4.95E-04**
		Simple mode		0.85 (0.69-1.04)	0.12
		Weighted mode		0.85 (0.70-1.02)	0.09
	*Ruminococcusgnavusgroup*	MR Egger	63	0.84 (0.58-1.21)	0.35
		Weighted median		0.80 (0.74-0.87)	**1.17E-07**
		Simple mode		0.79 (0.64-0.97)	**0.03**
		Weighted mode		0.80 (0.66-0.96)	**0.02**
	*Euryarchaeota*	MR Egger	44	0.82 (0.67-1.01)	0.07
		Weighted median		0.86 (0.80-0.92)	**1.35E-05**
		Simple mode		0.82 (0.69-0.97)	**0.02**
		Weighted mode		0.82 (0.69-0.97)	**0.02**
	*Gammaproteobacteria*	MR Egger	58	1.27 (0.72-2.26)	0.41
		Weighted median		1.12 (0.99-1.26)	0.08
		Simple mode		1.18 (0.87-1.59)	0.28
		Weighted mode		1.17 (0.87-1.55)	0.30
	*Eubacteriumeligensgroup*	MR Egger	12	1.72 (0.85-3.47)	0.16
		Weighted median		1.36 (1.06-1.76)	**0.02**
		Simple mode		1.48 (1.00-2.19)	0.08
		Weighted mode		1.48 (1.01-2.16)	0.07
	*Intestinimonas*	MR Egger	52	1.28 (0.96-1.71)	0.10
		Weighted median		0.93 (0.84-1.04)	0.22
		Simple mode		0.93 (0.75-1.16)	0.53
		Weighted mode		0.93 (0.75-1.15)	0.53
ECNEH	*CandidatusSoleaferrea*	MR Egger	41	0.41 (0.12-1.40)	0.16
		Weighted median		0.73 (0.59-0.90)	**3.77E-03**
		Simple mode		0.73 (0.47-1.13)	0.16
		Weighted mode		0.73 (0.48-1.10)	0.14
	*Ruminococcusgnavusgroup*	MR Egger	63	0.57 (0.23-1.38)	0.22
		Weighted median		0.43 (0.36-0.51)	**4.44E-20**
		Simple mode		0.41 (0.27-0.62)	**1.08E-04**
		Weighted mode		0.41 (0.27-0.62)	**9.24E-05**
	*Euryarchaeota*	MR Egger	44	0.58 (0.35-0.95)	**0.04**
		Weighted median		0.76 (0.64-0.90)	**1.77E-03**
		Simple mode		0.59 (0.40-0.86)	**0.01**
		Weighted mode		0.59 (0.40-0.86)	**0.01**
	*Gammaproteobacteria*	MR Egger	58	0.14 (0.03-0.58)	0.09
		Weighted median		2.57 (1.89-3.49)	0.19
		Simple mode		2.83 (1.42-5.66)	0.46
		Weighted mode		2.83 (1.42-5.67)	0.47
	*Eubacteriumeligensgroup*	MR Egger	12	13.34 (2.11-84.17)	**0.02**
		Weighted median		1.49 (0.73-3.06)	0.28
		Simple mode		2.88 (0.55-14.96)	0.23
		Weighted mode		2.81 (0.58-13.60)	0.23
	*Intestinimonas*	MR Egger	52	1.01 (0.51-2.01)	0.98
		Weighted median		1.94 (1.47-2.57)	**3.60E-06**
		Simple mode		1.96 (1.03-3.74)	0.05
		Weighted mode		1.96 (0.98-3.93)	0.06

No. SNP, the number of SNPs being used as instrumental variables; MR, Mendelian randomization; EC, endometrial cancer; ECEH, endometrial cancer with endometrioid histologies; ECNEH, endometrial cancer with non-endometrioid histologies; OR, odds ratio; CI, confidence interval.

**Table 3:** The heterogeneity results from Cochran's Q test

Traits (outcome)	Bacterial taxa (exposure)	Q	*P* value
EC	*CandidatusSoleaferrea*	8.38	1.00
	*Ruminococcusgnavusgroup*	44.78	0.95
	*Euryarchaeota*	36.37	0.75
	*Gammaproteobacteria*	29.90	1.00
ECEH	*CandidatusSoleaferrea*	14.61	1.00
	*Ruminococcusgnavusgroup*	61.09	0.51
	*Euryarchaeota*	28.54	0.96
ECNEH	*CandidatusSoleaferrea*	9.78	1.00
	*Ruminococcusgnavusgroup*	23.04	1.00
	*Euryarchaeota*	28.61	0.95
	*Intestinimonas*	50.16	0.51

EC, endometrial cancer; ECEH, endometrial cancer with endometrioid histologies; ECNEH, endometrial cancer with non-endometrioid histologies.

**Table 4:** Directional pleiotropy results from MR Egger intercept analysis

Traits (outcome)	Bacterial taxa (exposure)	MR Egger_intercept	SE	*P* value
EC	*CandidatusSoleaferrea*	-0.0072	0.0234	0.7598
	*Ruminococcusgnavusgroup*	-0.0051	0.0150	0.7342
	*Euryarchaeota*	-0.0047	0.0135	0.7304
	*Gammaproteobacteria*	0.0015	0.0151	0.9204
ECEH	*CandidatusSoleaferrea*	-0.0054	0.0274	0.8438
	*Ruminococcusgnavusgroup*	-0.0038	0.0177	0.8319
	*Euryarchaeota*	0.0167	0.0160	0.3027
ECNEH	*CandidatusSoleaferrea*	0.0660	0.0661	0.3237
	*Ruminococcusgnavusgroup*	-0.0117	0.0432	0.7875
	*Euryarchaeota*	0.0431	0.0392	0.2772
	*Intestinimonas*	0.0240	0.0290	0.4126

EC, endometrial cancer; ECEH, endometrial cancer with endometrioid histologies; ECNEH, endometrial cancer with non-endometrioid histologies; MR, Mendelian randomization.

## Abbreviations

EC: Endometrial cancer
PCOS: polycystic ovarian syndrome
GUS: glucuronidase
MR: Mendelian randomization
SNPs: single nucleotide polymorphisms
IVs: instrumental variables
GWAS: Genome-wide Association Study
mbQTL: microbiota quantitative trait locus
LD: linkage disequilibrium
MR-PRESSO: MR pleiotropy residual sum and outlier
IVW: inverse-variance weighted
InSIDE: Instrument Strength Independent of Direct Effect
LPS: Lipopolysaccharide
TMAO: trimethylamine N-oxide
PAMP: pathogen-associated molecular pattern
PGE2: prostaglandin E2
EMT: epithelial-mesenchymal transition
VEGFA: vascular endothelial growth factor A
T2DM: type 2 diabetes mellitus
GLP-2: glucagon-like peptide 2
SCFAs: short-chain fatty acids
IR: insulin resistance
TMA: trimethylamine
