# Supplementary material for: Causal Relationship between Gut Microbiota and Endometrial Cancer: A Two-Sample Mendelian Randomization Study
Source: Int J Med Sci. 2025 Jun 23;22(12):3142–53. doi: 10.7150/ijms.112922 (PMC12244081; doi:10.7150/ijms.112922)
Supplement: Supplementary file 1 — Supplementary figures. [file ijmsv22p3142s1.pdf]

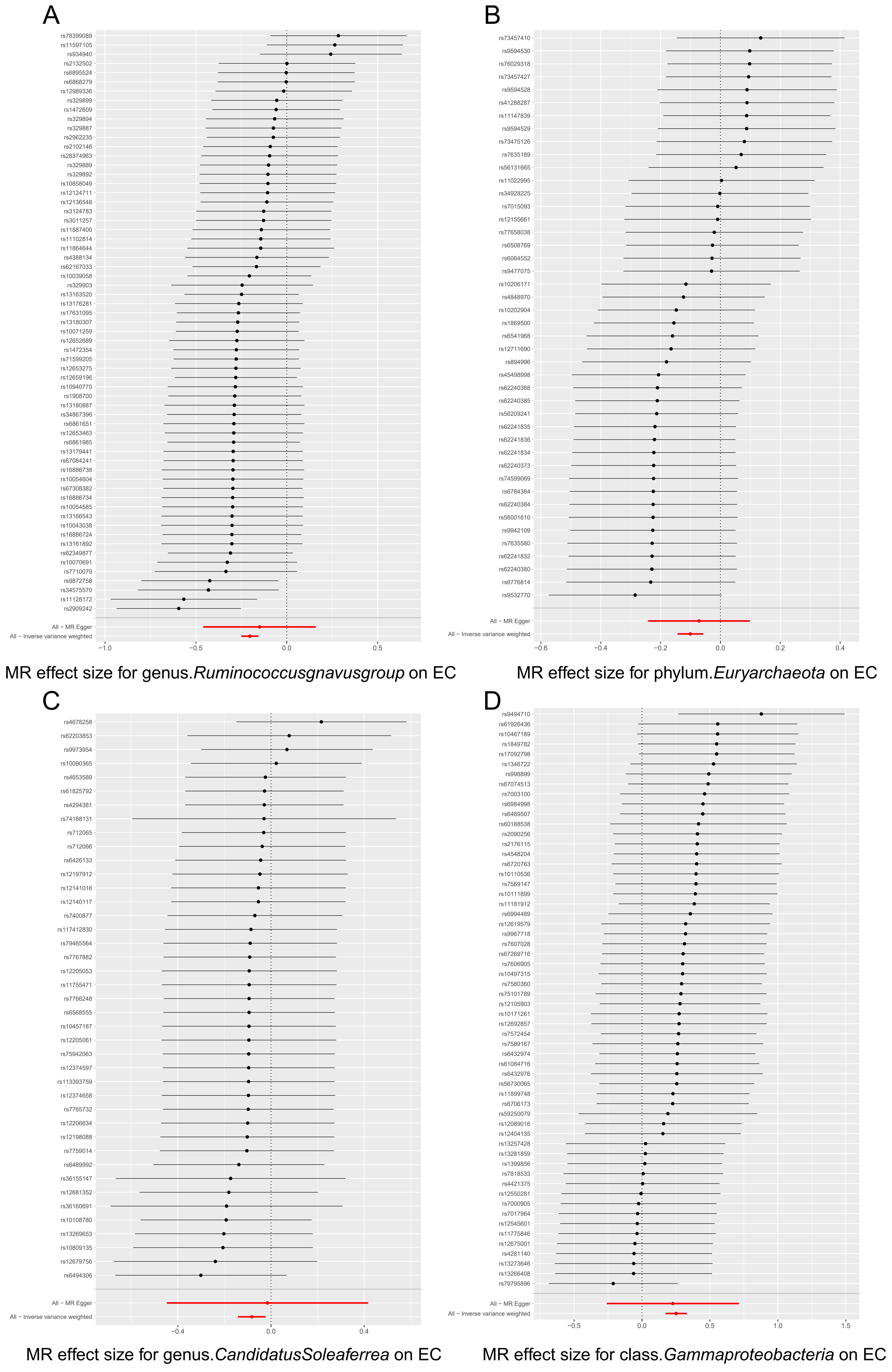
A
B
C
D
All − MR Egger
All − Inverse variance weighted
MR effect size for genus.*Ruminococcusgnavusgroup* on EC
MR effect size for phylum.*Euryarchaeota* on EC
MR effect size for genus.*CandidatusSoleaferrea* on EC
MR effect size for class.*Gammaproteobacteria* on EC

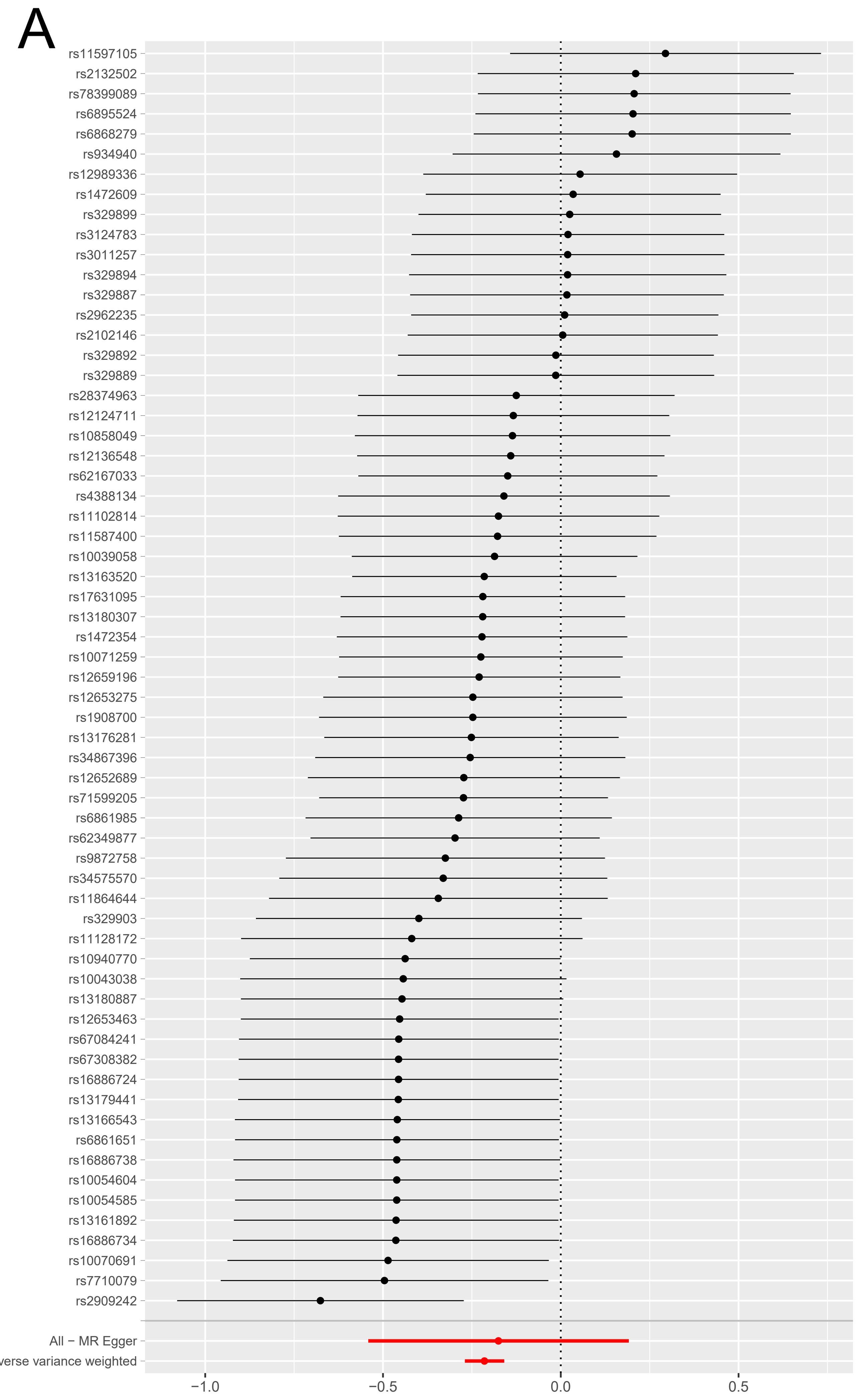

MR effect size for genus.*Ruminococcusgnavusgroup* on ECEH

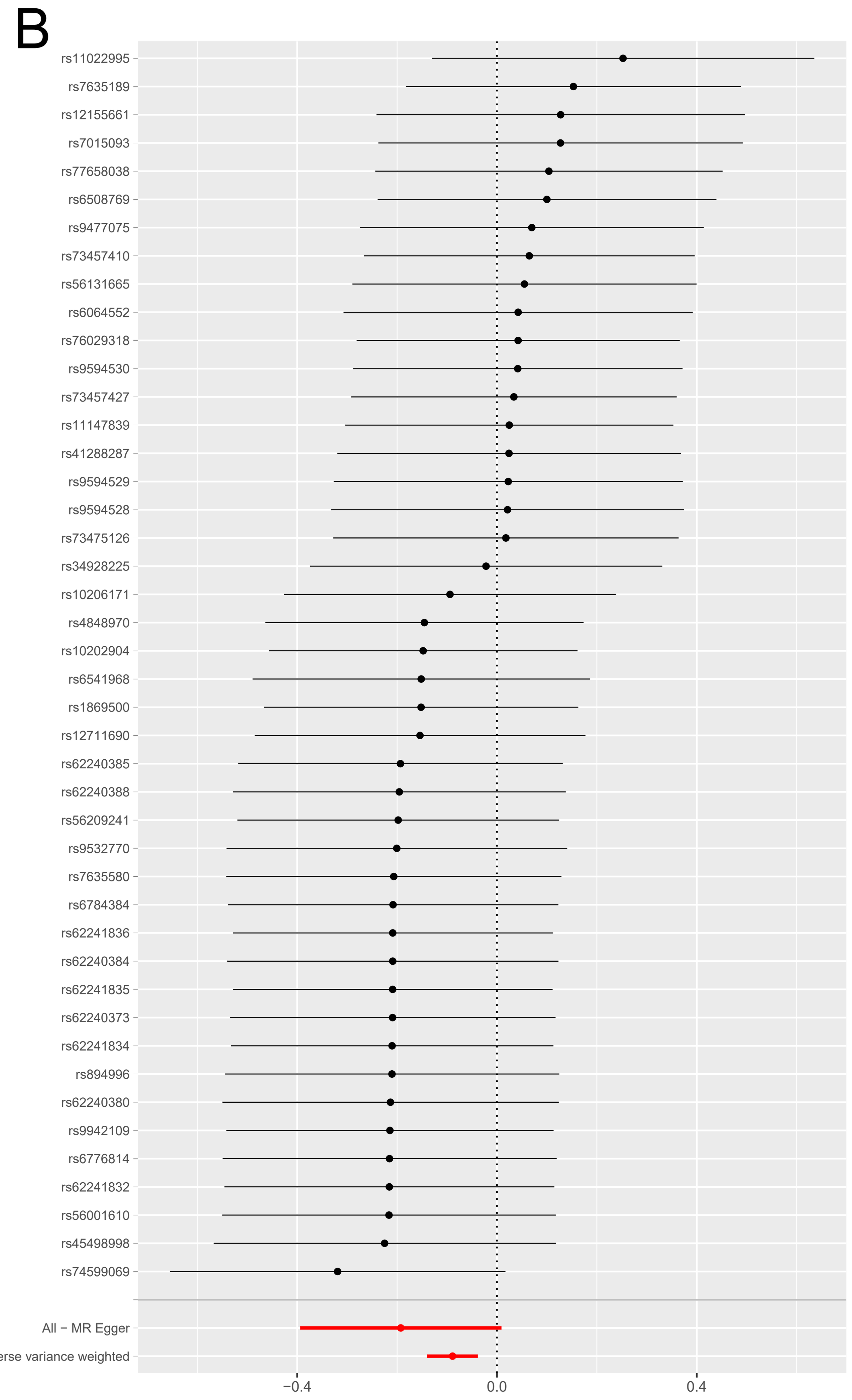

MR effect size for phylum.*Euryarchaeota* on ECEH

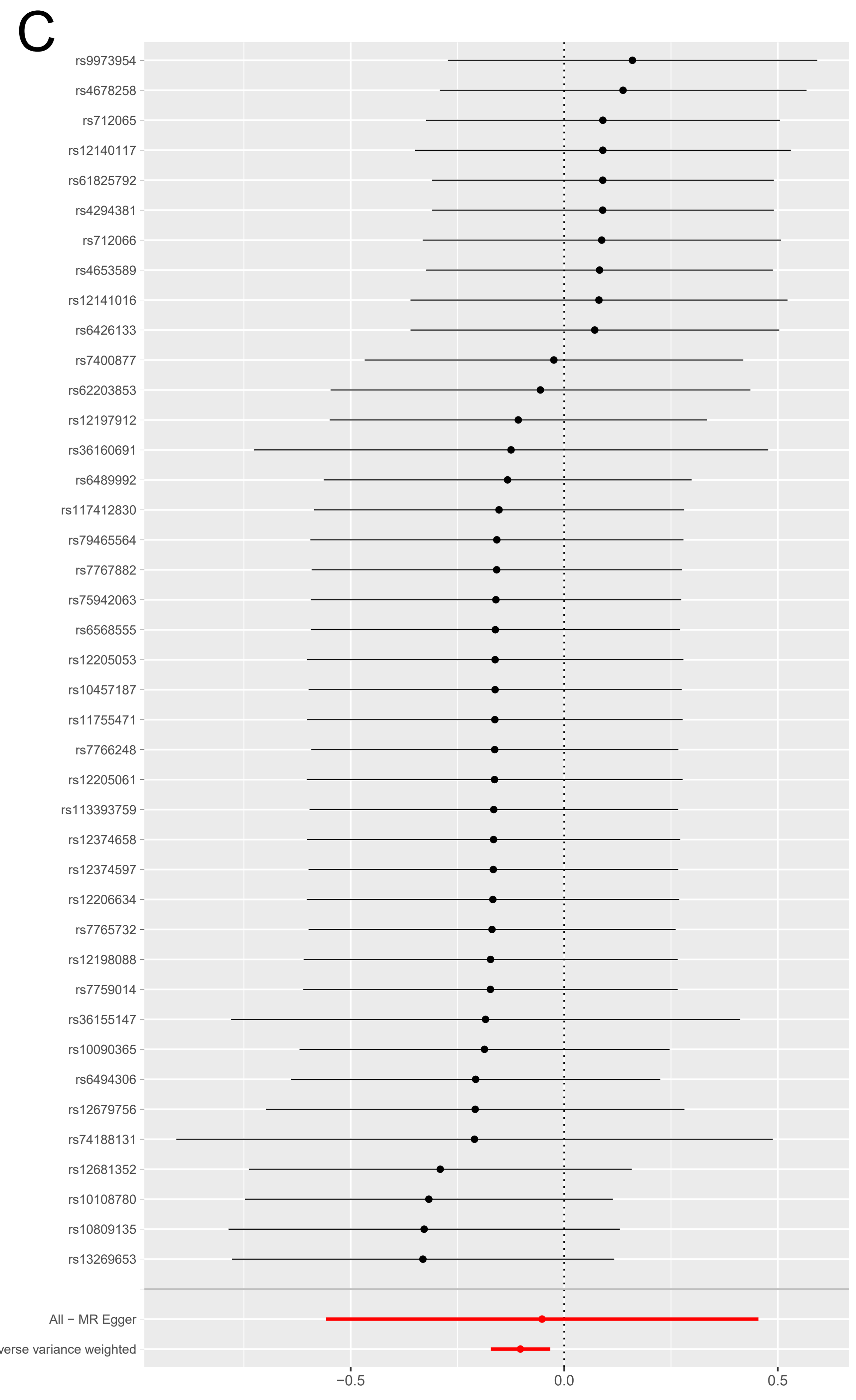

MR effect size for genus.*CandidatusSoleaferrea* on ECEH

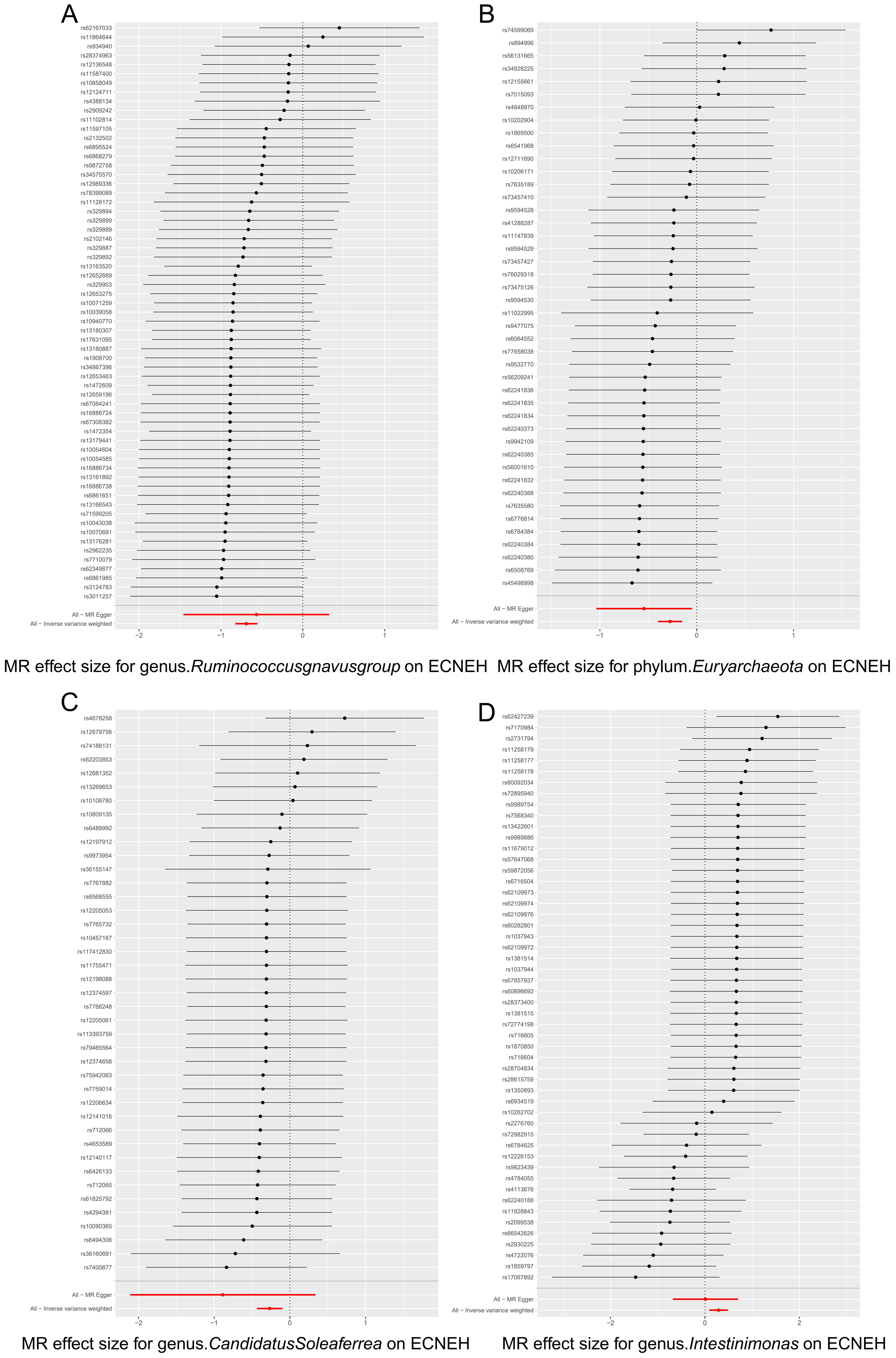
A
All − MR Egger
All − Inverse variance weighted
MR effect size for genus.*Ruminococcusgnavusgroup* on ECNEH
B
All − MR Egger
All − Inverse variance weighted
MR effect size for phylum.*Euryarchaeota* on ECNEH
C
All − MR Egger
All − Inverse variance weighted
MR effect size for genus.*CandidatusSoleaferrea* on ECNEH
D
All − MR Egger
All − Inverse variance weighted
MR effect size for genus.*Intestinimonas* on ECNEH

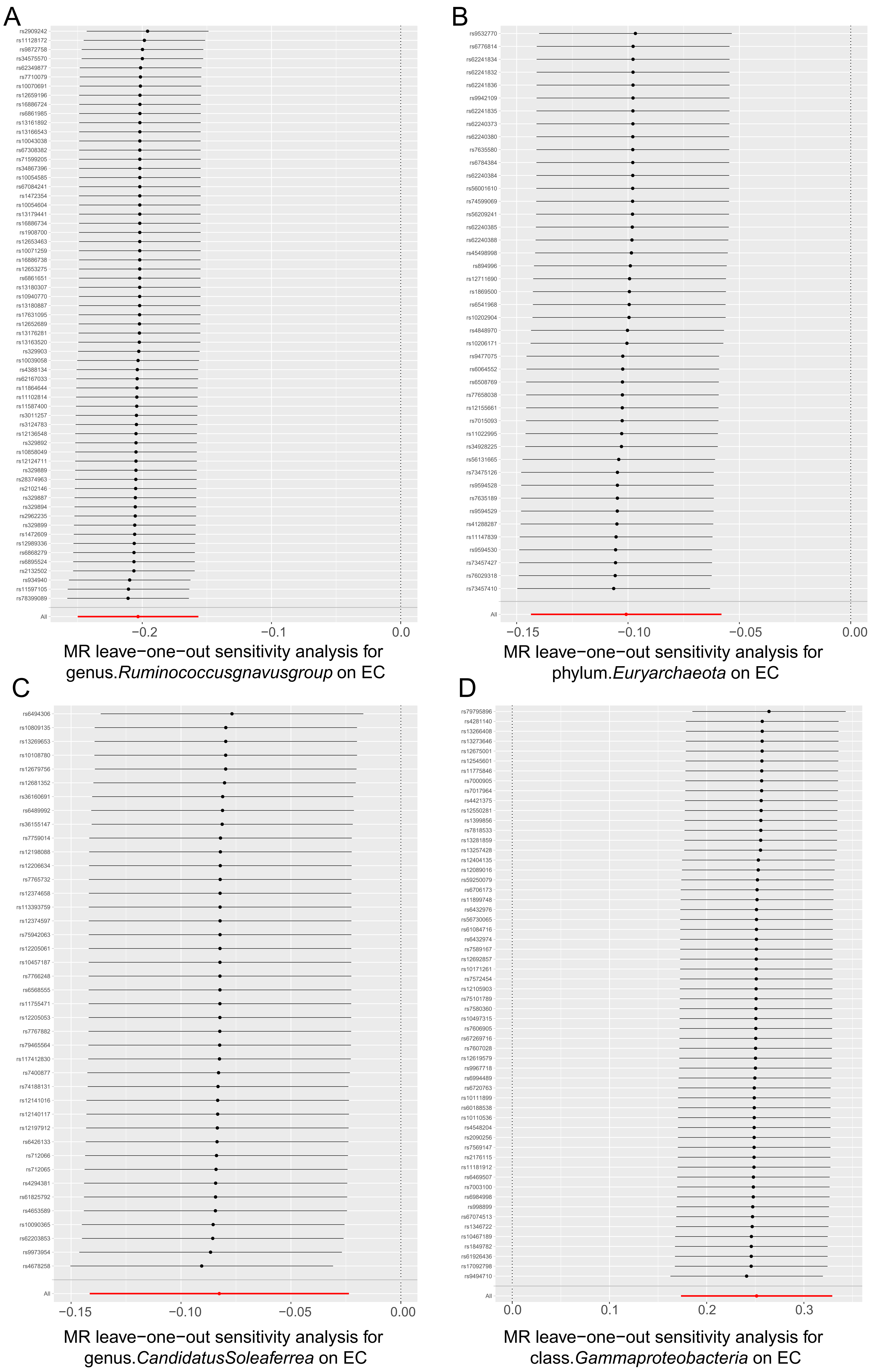
A
−0.2
−0.1
0.0
MR leave−one−out sensitivity analysis for
genus.*Ruminococcusgnavusgroup* on EC
B
−0.15
−0.10
−0.05
0.00
MR leave−one−out sensitivity analysis for
phylum.*Euryarchaeota* on EC
C
−0.15
−0.10
−0.05
0.00
MR leave−one−out sensitivity analysis for
genus.*CandidatusSoleaferrea* on EC
D
0.0
0.1
0.2
0.3
MR leave−one−out sensitivity analysis for
class.*Gammaproteobacteria* on EC

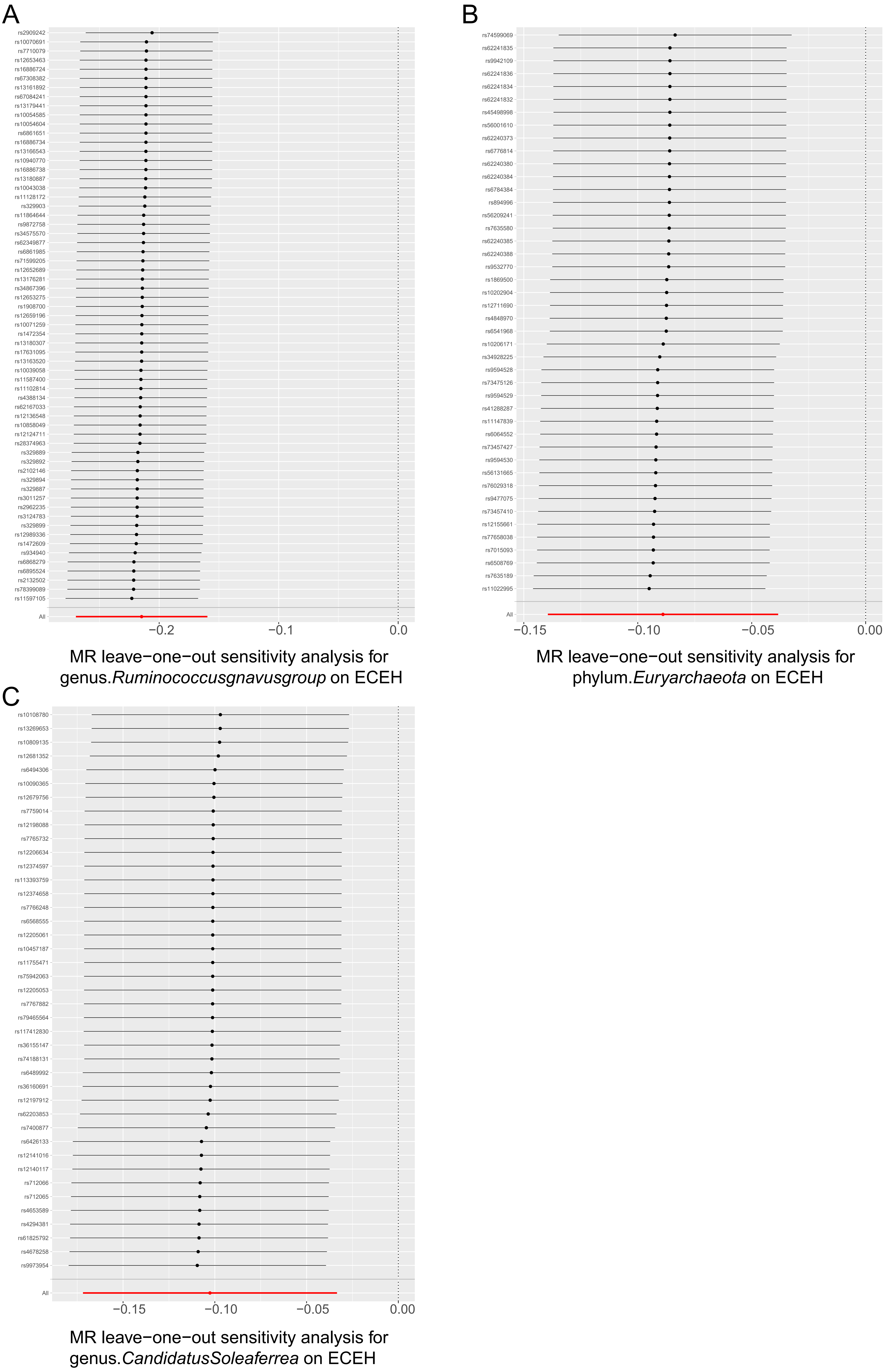
A
−0.2
−0.1
0.0
All
MR leave−one−out sensitivity analysis for
genus.*Ruminococcusgnavusgroup* on ECEH
B
−0.15
−0.10
−0.05
0.00
All
MR leave−one−out sensitivity analysis for
phylum.*Euryarchaeota* on ECEH
C
−0.15
−0.10
−0.05
0.00
All
MR leave−one−out sensitivity analysis for
genus.*CandidatusSoleaferrea* on ECEH

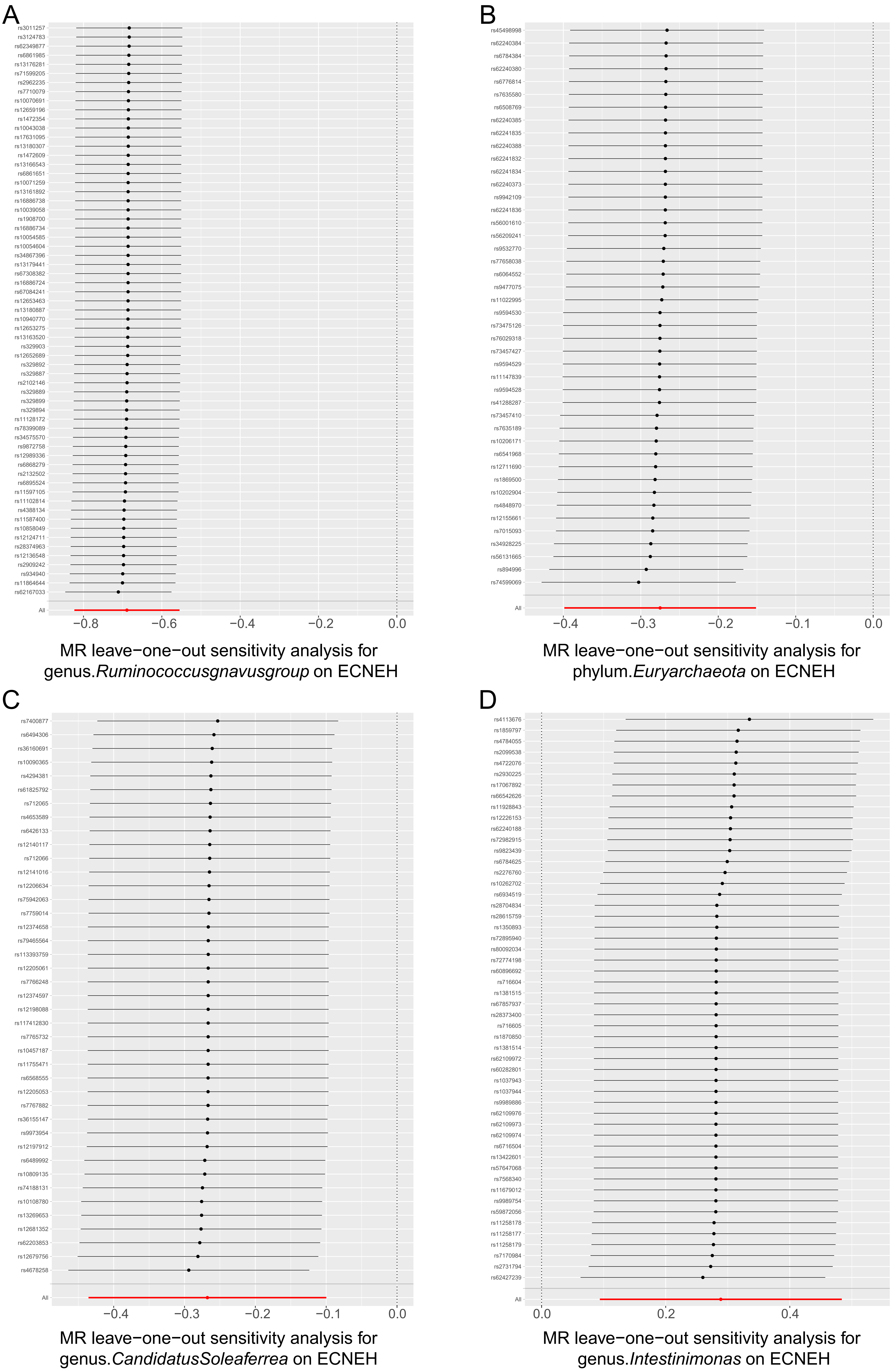
A
All
−0.8
−0.6
−0.4
−0.2
0.0
MR leave−one−out sensitivity analysis for genus.Ruminococcusgnavusgroup on ECNEH
B
All
−0.4
−0.3
−0.2
−0.1
0.0
MR leave−one−out sensitivity analysis for phylum.Euryarchaeota on ECNEH
C
All
−0.4
−0.3
−0.2
−0.1
0.0
MR leave−one−out sensitivity analysis for genus.CandidatusSoleaferrea on ECNEH
D
All
0.0
0.2
0.4
MR leave−one−out sensitivity analysis for genus.Intestinimonas on ECNEH

Supplementary Figure 1 Diagnostic power of SNPs of exposure factors on EC. (**A**) MR effect of SNPs of *Ruminococcusgnavusgroup* on EC. (**B**) MR effect of SNPs of *Euryarchaeota* on EC. (**C**) MR effect of SNPs of *CandidatusSoleaferrea* on EC. (**D**) MR effect of SNPs of *Gammaproteobacteria* on EC. MR, Mendelian randomization; EC, endometrial cancer; SNP, single nucleotide polymorphism.

Supplementary Figure 2 Diagnostic power of SNPs of exposure factors on ECEH. (**A**) MR effect of SNPs of *Ruminococcusgnavusgroup* on ECEH. (**B**) MR effect of SNPs of *Euryarchaeota* on ECEH. (**C**) MR effect of SNPs of *CandidatusSoleaferrea* on ECEH. MR, Mendelian randomization; ECEH, endometrial cancer with endometrioid histology; SNP, single nucleotide polymorphism.

Supplementary Figure 3 Diagnostic power of SNPs of exposure factors on ECNEH. (**A**) MR effect of SNPs of *Ruminococcusgnavusgroup* on ECNEH. (**B**) MR effect of SNPs of *Euryarchaeota* on ECNEH. (**C**) MR effect of SNPs of *CandidatusSoleaferrea* on ECNEH. (**D**) MR effect of SNPs of *Intestinimonas* on ECNEH. MR, Mendelian randomization; ECNEH, endometrial cancer with non-endometrioid histology; SNP, single nucleotide polymorphism.

Supplementary Figure 4 Leave-one-out analysis of the causal impact of gut microbiota on EC. (**A**) Leave-one-out analysis of the causal impact of *Ruminococcusgnavusgroup* on EC. (**B**) Leave-one-out analysis of the causal impact of *Euryarchaeota* on EC. (**C**) Leave-one-out analysis of the causal impact of *CandidatusSoleaferrea* on EC. (**D**) Leave-one-out analysis of the causal impact of *Gammaproteobacteria* on EC. MR, Mendelian randomization; EC, endometrial cancer.

Supplementary Figure 5 Leave-one-out analysis of the causal impact of gut microbiota on ECEH. (**A**) Leave-one-out analysis of the causal impact of *Ruminococcusgnavusgroup* on ECEH. (**B**) Leave-one-out analysis of the causal impact of *Euryarchaeota* on ECEH. (**C**) Leave-one-out analysis of the causal impact of *CandidatusSoleaferrea* on ECEH. MR, Mendelian randomization; ECEH, endometrial cancer with endometrioid histology.

Supplementary Figure 6 Leave-one-out analysis of the causal impact of gut microbiota on ECNEH. (**A**) Leave-one-out analysis of the causal impact of *Ruminococcusgnavusgroup* on ECNEH. (**B**) Leave-one-out analysis of the causal impact of *Euryarchaeota* on ECNEH. (**C**) Leave-one-out analysis of the causal impact of *CandidatusSoleaferrea* on ECNEH. (**D**) Leave-one-out analysis of the causal impact of *Intestinimonas* on ECNEH. MR, Mendelian randomization; ECNEH, endometrial cancer with non-endometrioid histology.
